# Endometrial scratching in unexplained repeated implantation failure causes two competing forces, angiogenesis and anti-angiogenesis: An RCT study

**DOI:** 10.18502/ijrm.v22i4.16387

**Published:** 2024-06-12

**Authors:** Samaneh Aghajanpour, Fereshteh Mehraein, Fatemehsadat Amjadi, Zahra Zandieh, Firouzeh Ghaffari, Khashayar Aflatoonian, Elham Hosseini, Mehrdad Bakhtiyari, Reza Aflatoonian

**Affiliations:** ^1^Department of Anatomy, School of Medicine, Iran University of Medical Sciences, Tehran, Iran.; ^2^Minimally Invasive Surgery Research Center, Iran University of Medical Sciences, Tehran, Iran.; ^3^Reproductive Sciences and Technology Research Center, Department of Anatomy, School of Medicine, Iran University of Medical Sciences, Tehran, Iran.; ^4^Department of Endocrinology and Female Infertility, Reproductive Biomedicine Research Center, Royan Institute for Reproductive Biomedicine, ACECR, Tehran, Iran.; ^5^School of Medicine, Iran University of Medical Sciences, Tehran, Iran.; ^6^Department of Obstetrics and Gynecology, Mousavi Hospital, School of Medicine, Zanjan University of Medical Sciences, Zanjan, Iran.; ^7^Zanjan Metabolic Diseases Research Center, Zanjan University of Medical Sciences, Zanjan, Iran.

**Keywords:** Endometrium, Angiogenesis, Embryo implantation, Polymerase chain reaction.

## Abstract

**Background:**

A significant association between endometrial vascularity and pregnancy has been shown in previous research, while poor vascularization was attributed to repeated implantation failure (RIF). One possible approach to enhance angiogenesis for successful implantation is endometrial scratching (ES).

**Objective:**

The purpose was to investigate endometrial responses to scratching by profiling angiogenesis-related gene expression in unexplained RIF participants.

**Materials and Methods:**

In this randomized controlled trial study, 20 infertile women with unexplained RIF were assigned to 2 groups by the balanced block randomization method (n = 10/each group): the intervention group (group A) (who received ES in the follicular phase) and the control group (group B). Endometrial biopsy was performed in the secretory phase. Gene expression profiling was performed using a polymerase chain reaction-array kit for human-angiogenic growth factors. The implantation and clinical pregnancy rates were also assessed.

**Results:**

Among the angiogenesis-promoting genes, *FGF1*, *FGF13*, *FGF2*, *TGFA*, *ANG*, *ANGPT1*, and *VEGFA* were significantly upregulated (p < 0.05). *IL12A* (an angiogenesis-inhibiting cytokine) was significantly upregulated (p < 0.01). In contrast, 15 genes with angiogenesis-related functions, including *CXCL11*, *CXCL13*, *CXCL3*, *CXCL5*, *CXCL6*, *EREG*, *FIGF*, *FST*, *IL10*, *LEP*, *PPBP*, *PROK1*, *RHOB*, *TNF*, and *TYMP, *were downregulated after ES. No significant differences were observed between the intervention (group A) and control (group B) groups in terms of implantation (43.75% vs. 28.57%) or clinical pregnancy rates (75% vs. 57.1%).

**Conclusion:**

ES induced significant alterations in the expression of angiogenesis-related genes, with notable up/downregulation of key angiogenic/antiangiogenic factors. These findings enhance our understanding of the molecular responses triggered by ES, underscoring the potential influence of ES on the complex processes of angiogenesis crucial for implantation.

## 1. Introduction

During the window of implantation (WOI), endometrial cells of the uterus undergo dynamic morphological and functional differentiation to provide a nourishing milieu (1, 2). Inadequate endometrial angiogenesis during this period can interfere with optimal endometrial receptivity, resulting in repeated implantation failure (RIF), which is the greatest clinical hurdle to overcome following the in vitro fertilization (IVF)-embryo transfer (ET) procedure (3).

In the endometrium, normal angiogenesis occurs repeatedly during both the menstrual cycle and during embryo implantation (4). Sufficient endometrial angiogenesis is mediated by various cytokines and growth factors that promote spiral arterial remodeling. Alterations in these factors have been proven in women with RIF (3, 5). Previous studies have shown a robust correlation between endometrial vascularity and an improved pregnancy rate. In contrast, poor vascularization of endometrial cells was linked to an increased risk of miscarriage and RIF (6, 7).

Despite the importance of angiogenesis in successful implantation and maintenance of pregnancy, there is currently no effective evidence-based potential therapy for preventing or treating this condition. One of the suggested approaches is endometrial scratching (ES) before ET (8, 9). ES is proposed to enhance endometrial preparation for embryo implantation through various mechanisms. This procedure is believed to induce a controlled local inflammatory response within endometrial tissue, creating an environment that fosters the release of cytokines and growth factors, ultimately improving endometrial receptivity (10, 11). Additionally, ES may stimulate angiogenesis, promoting the formation of new blood vessels in the endometrium and thereby establishing a more supportive environment for successful embryo implantation. Furthermore, the procedure is thought to modulate the local immune response, influencing immune cell activity and cytokine release to create a microenvironment conducive to embryo implantation (12).

Although ES appears to be less effective in nonselected infertile women, it positively affects the implantation rate in women who have experienced RIF (13, 14). Therefore, ES presents several controversial issues that contribute to ongoing debates in the field. Another contentious aspect is the optimal timing and frequency of ES, with debates surrounding whether a single or multiple scratching procedures provide superior outcomes and whether conducting the procedure during the cycle preceding IVF consistently enhances implantation rates. The delicate balance between potential harm and benefit is also a subject of disagreement, as some studies suggest a transient inflammatory response that may positively influence endometrial receptivity, while others express concerns about potential harm, including uterine scarring or adverse effects on the endometrial microenvironment (10, 11).

These controversies underscore the need for further research and a nuanced understanding of the multifaceted aspects of ES in infertility treatment. Given the unclear understanding of the changes induced by ES in the endometrium, we conducted an analysis focused on profiling angiogenesis-related gene expression. This approach aimed to shed light on the underlying physiology influenced by ES and its potential impact on pregnancy rates.

## 2. Materials and Methods

### Study design and participants

This randomized, double-blind controlled trial was conducted on 20 infertile women with unexplained RIF (uRIF) admitted to Laleh hospital, Tehran, Iran between June 2021 and January 2023.

### Blinding

The study was a double-blind study (blinded participants/blinded researcher). Before block randomization, sealed and numbered envelopes were used to hide the treatment allocation. The endometrial specimens from both groups were transferred to the research laboratory, where neither the technicians nor the researchers were aware of the treatment assignments; also, all study-related report forms documented the randomization code (researcher blindness).

### Inclusion criteria

Women were considered eligible for enrollment in the research if they had uRIF (unknown definite cause of RIF) and failed to conceive after 3 or more ET cycles using high-quality transferred embryos (at least 1 blastocyst ET cycle). Other common inclusion criteria, including age 20–40 yr, body mass index < 25 kg/m^2^, good response to stimulation of previous ovulation, having at least 2 embryos with a good grade, and normal results of hysterosalpingography or hysteroscopy, were considered for enrollment in the RCT.

### Exclusion criteria

The exclusion criteria were as follows: the thickness of the endometrium on the day of human chorionic gonadotropin (hCG) hormone injection was < 7 mm; uterine conditions associated with RIF (congenital malformations, intramural and subserousal myoma [ > 5 cm]), submucosal myoma, endometrioma ( ≥ 3 cm), adhesions, hydrosalpinges, uterine or ovarian surgery, severe male factor infertility (males with testicular sperm extraction, sperm freezing, and a DNA fragmentation index of sperm ≥ 16%); fewer than 2 available embryos in the present study; and endometrial tuberculosis or previous treatment for tuberculosis.

Participants with a history of diabetes, thyroid disease, any endocrine, genetic, infection, autoimmune disorders, other hormonal diseases, abnormal preimplantation genetic test results, or discomfort during endometrial sampling due to severe pain or failure to return to prepare an endometrial sample were also excluded from the study.

**Figure 1:**
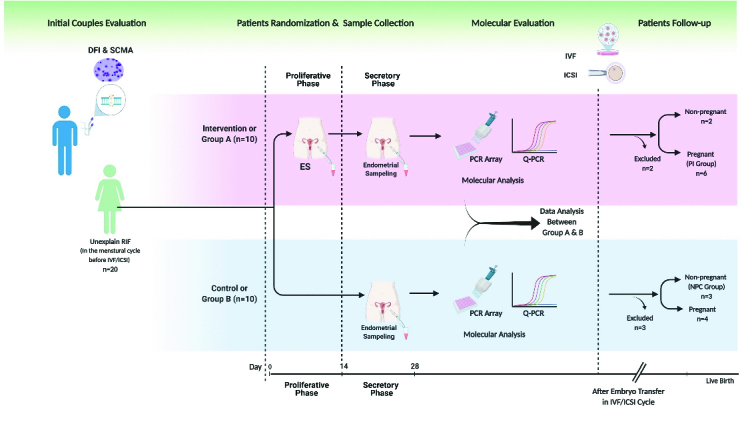
An overview of how participants were randomized into study groups for molecular investigation. Created with BioRender.com.

For this study, 168 medical files of RIF participants in Laleh hospital, Tehran, Iran were evaluated. We included only individuals with uRIF, and other causes of RIF were not considered. The study continued with a total of 20 women who were randomly assigned to 2 groups, the intervention group (group A) or the control group (group B), using the permuted block randomization method (n = 10/each group). An epidemiologist, utilizing STATA 13.0 software (STATA corp, college station, TX), implemented the block randomization approach with a total of 5 blocks, each consisting of 4 participants. Before block randomization, sealed and numbered envelopes were utilized to conceal the treatment allocation. A nurse, on the day of the procedure, removed the envelope just before the participant entered the surgery room and assigned them to either the intervention (group A) or control group (group B). Figure 1 shows how participants were randomized into study groups for molecular investigation.

### Sample size

The sample size was determined using G*Power version 3.1.9.4 software based on the difference in gene expression, which was the primary outcome investigated in a previous report (10). Based on a type I error rate of 5% (α = 0.05), a power of 80%, and a type II error rate of 0.20, the sample size was calculated to be 20 (10 in each group). In this study, considering the effect size d = 0.9800000 and using the formula: 


$$n_{B}=n_{A}\to \;n_{A}=\frac{2{{(Z}_{1-\alpha /2}+Z_{1-\beta })}^{2}\sigma^{2}}{d^{2}}$$

The number of samples (10 in each group) were determined. In the formula, n is the number of samples, d is the effect size, sigma is the standard deviation, z1-b is the test power of 80%, and z1-a/2 is 1.96.

A researcher midwife in the clinic enrolled participants, assigned them to the control (group B) and intervention (group A) groups, and counseled them about the nature of the study. On days 8–11 of the menstrual cycle, a designated gynecologist performed ES in the intervention group (group A). An endometrial injury was assessed by the same physician during the secretory phase of the same cycle on days 19–23. On the day of scratching (days 8–11), participants in the control group (group B) were referred to the clinic, and all treatments were conducted except for scratching.

### Evaluation of chromatin and DNA status of spermatozoa

In addition to routine semen analysis, the semen of each selected couple was evaluated with sperm chromatin dispersion and aniline blue tests to rule out the effects of sperm DNA fragmentation on embryo development and implantation. Sperm chromatin dispersion test was performed using a sperm DNA fragmentation kit (SDFA Kit, Ideh Varzan Farda, Iran) according to the manufacturer's instructions. The differentiation between normal sperm and borderline or abnormal sperm in terms of DNA status is determined by a threshold value below 15% for the DNA fragmentation index (15). At this cut-off rate, sperm with a large or medium halo is classified as having intact chromatin. In contrast, those with a small or no halo (thickness equal to or smaller than 1/3 diameter of the minor diameter of the core) were classified as having fragmented DNA.

The degree of sperm chromatin compaction is evaluated using aniline blue staining, which detects chromatin abnormalities in sperm nuclei associated with their nucleoprotein content and DNA. In this method, a sperm chromatin maturation assay kit (SCMA Kit, Ideh Varzan Farda., Iran) was used to differentiate between lysine-rich histones and protamine-rich sperm nuclei. A percentage of immature sperm showing less than 20% histone-rich (stained blue) versus mature protamine-rich nuclei were classified as normal (16).

### ES procedure

ES was performed in the intervention group (group A) during the proliferative phases (days 9–11), followed by a biopsy obtained during the luteal phases (days 19–21) of the cycle preceding ovarian stimulation. In the control group (group B), participants underwent a similar procedure involving vaginal speculum placement during the proliferative phase at an outpatient appointment. A soft catheter was introduced without the scratching action to maintain procedural consistency to ensure a similar procedural experience for the control group (group B). Biopsy samples were obtained from both groups during the luteal phases (days 19–21) of the cycle preceding ovarian stimulation.

To scratch the uterine endometrium, a 3 mm wide catheter (Pipelle; Gynetics Medical Products, Belgium) was introduced via the cervix and into the uterine cavity. It was then rotated and moved back and forth. Once placed in the uterus, the flexible Pipelle must be rotated approximately 360 degrees and then relocated up and down 4 times (10). Later, endometrial tissues were subsequently preserved in RNA and cryopreserved at -80 C for genomic analysis.

### Ovarian stimulation protocol and intracytoplasmic sperm injection (ICSI)

An agonist ovarian stimulation protocol was used for all infertile women. Briefly, subcutaneous injections of 500 mg of suprefact (Buserelin, SANOFI, Germany) per day for 14 days beginning on days 19–21 of the menstrual cycle were used for pituitary suppression, followed by daily administration of 150–225 IU of gonadotropin (Cinnal-F, Follitropinalfa, CinnaGen, Iran) for ovarian stimulation and Ovitrelle (500 mg, Choriogonadotropinalfa, MERCK, Switzerland) for final oocyte triggering. The dose of follitropinalfa was justified by the woman's age, antral follicle count, and anti-Müllerian hormone (AMH) level. Baseline AMH levels (ELISA Kit, DiaZist, Iran), follicle-stimulating hormone (Architect FSH Reagent Kit, Abbott, USA), and luteinizing hormone (Architect LH Reagent Kit, Abbott, USA) were measured.

The total dose of gonadotropin was measured based on the number of IUs received during the ovarian stimulation cycle, based on the timeline from the beginning of ovarian stimulation to the time of hCG injection. The duration of ovarian stimulation was defined as the number of days between the start of gonadotropin administration and the start of hCG injection (the number of days the participant received the medicine).

After the pick-up procedure, oocyte denudation was carried out, followed by ICSI on metaphase II oocytes, identified by the presence of the first polar body. The next day after sperm injection, the number of oocytes with 2 pronuclei was observed and divided by the total number of injected oocytes to determine the fertilization rate.

The embryo development rate was calculated as the number of embryos on day 3 divided by the number of fertilized oocytes (with 2 pronuclei).

The quality of the embryos was assessed by i) the number of blastomeres on the 2 ${\;}^{\text{nd}}$ and 3 ${\;}^{\text{rd}}$ days after ICSI, ii) the absence of multinucleotide blastomeres, and iii) the percentage of fragmented blastomeres. High-quality embryos (stage-specific cell size, no multinucleation, or fragmentation < 10%) were transferred into the uterine cavity, followed by intramuscular progesterone injection (ProgestinⓇ, Aburaihan Pharmaceutical Co., Iran) for secretory phase support. β-human-derived chorionic gonadotrophic hormone (βhCG) levels were measured 14 days after ET. 5 wk later, the gestational sac and fetal heart rate were assessed via vaginal ultrasonography in the positive cases (17, 18).

Chemical pregnancy was measured based on the βhCG titer 2 wk after ET. A blighted ovum was observed 5 wk after ET, defined as the absence of a fetus in the gestational sac according to an ultrasound report. Ectopic pregnancy was determined 5 wk after ET, which was defined, according to the ultrasound report, as a gestational sac outside the uterus with a positive βhCG titer. Miscarriage was defined as pregnancy loss before wk 20, which was determined by the excretion of pregnancy remnants with vaginal bleeding (or the absence of a heartbeat according to the ultrasound report). Multiple pregnancies (twins) were determined 5 wk after ET according to an ultrasound report based on the number of gestational sacs ( < 1) with the embryo. Live birth was defined as the birth of a live baby at the time of delivery.

### Measuring outcomes

The main objective was to examine dynamic changes in angiogenic/antiangiogenic indicators of endometrial receptivity in response to scratching at the mRNA level. In addition, we evaluated clinical outcomes (implantation and clinical pregnancy rates) to determine the effect of ES on those clinical parameters. The implantation rate was determined by n ${\;}_{\text{gestational}\text{sacs}}$ /n ${\;}_{\text{transferred}\text{embryos}}$ 5 wk after ET. The presence of gestational sacs and fetal heartbeat on ultrasonography by using vaginal ultrasound 5 wk later was characterized as clinical pregnancy (19). The ET cancelation rate was calculated for participants with no ET. The chemical pregnancy rate was calculated based on the participants with βhCG titer per participant with ET.

The blighted ovum rate was calculated based on participants with the gestational sac without a fetus per participant with ET. The ectopic pregnancy rate was calculated for participants with a gestational sac outside the uterus and a positive βThCG titer per participant with ET. The miscarriage rate was calculated as participants with pregnancy loss per participant with ET. The multiple pregnancy rate was calculated based on the number of participants with more than one gestational sac with an embryo per participant with ET. The live birth rate was defined as the number of participants with the birth of a live baby per participant with ET. All pregnant women in both groups were monitored until delivery.

### Molecular analysis

The total RNA of endometrial tissue samples was extracted using TRIzol reagent (Sigma-Aldrich, USA), following the manufacturer's instructions. Subsequently, the purity and concentration of the total RNA samples were evaluated using a Nanodrop 2000 spectrophotometer (Thermo Scientific, USA). An RT2 first strand kit (Cat. No: 330404; Qiagen, Germany) was used for the removal of genomic DNA from the RNA samples and cDNA synthesis. A polymerase chain reaction (PCR) array Human Angiogenic Growth Factors Kit (Cat. No. 330231 PAHS-072ZA, Qiagen, Germany) and RT2 SYBR Green ROX qPCR master mix (Cat. No: 330523, Qiagen, Germany) were used to analyze a targeted panel of 84 genes related to angiogenesis via the step one plus real-time PCR system (Applied Biosystems, USA).

To create a table of Ct values, the acquired numbers were exported to an Excel file, which was then uploaded to the data analysis web portal at http://www.qiagen.com/geneglobe. C ${\;}_{\text{t}}$ values were normalized based on 3 manually selected reference genes (housekeeping gene). The web portal was utilized to compute the fold change/regulation using the ΔΔ Ct method. This process involved calculating Δ Ct between the gene of interest and the average of the housekeeping gene, followed by ΔΔ Ct calculations ( Δ Ct [case group]- Δ Ct [control]). Subsequently, the fold change was determined using the 2- ΔΔ Ct formula. The data analysis web portal also included volcano plots, scatter plots, and clustergrams.

Additionally, quantitative real-time PCR was performed on the 9 genes that showed the most significant changes. This method was conducted using Power SYBR Green Master Mix (Applied Biosystems, USA) to validate the results of the PCR array. The upregulated genes FGF1, IL12A, and VEGFA and the downregulated genes IL12B, IL17F, COL18A1, SERPINF1, TNFα, and CXCL11 were selected for confirmation. Moreover, 3 additional genes, vascular endothelial growth factor receptor 1 (VEGFR1), VEGFR2 (a marker of endometrial angiogenesis and a receptor for VEGF), and E-cadherin (a cell-cell adhesion molecule involved in endometrial receptivity), were also evaluated.

The sequences of the primers used in the present study are shown in table I. Human β -actin was used as a housekeeping gene, so the threshold cycle values (C ${\;}_{t}$ values) of the target genes were normalized to the threshold value of the reference gene. Melting curve analysis was used to confirm the specificity of the PCR amplification.

**Table 1:** Sequences of primers used for confirming array data by real-time PCR analysis


**Gene name**	**Forward primer**	**Reverse primer**	**Product size**
***COL18A1***	ACATCTCCCTGCTCTACACAGA	GCATTCTCTGGAACTCCTCACA	159
***IL12A***	CTAAAAAGCGAGGTCCCTCCAA	CTTCTTTCCCCCTCCCTAGTTC	102
***IL12B***	TGTGACACCCCTGAAGAAGATG	CTTAGAACCTCGCCTCCTTTGT	146
***IL17F***	CCAAGGCTGCTCTGTTTCTTTC	ACTGGGTAAGGAGTGGCATTTC	146
***FGF1***	CACCTGACCTCAACTAACCCTT	GGGCATTTTATAGGCATTGGGC	136
***CXCL11***	GGTAAAAGCAGTGAAAGTGGCA	GCTTCGATTTGGGATTTAGGCA	134
***SERPINF1***	AAGGGGCAGTGGGTAACAAA	GTAAAACAGCCTTAGGGTCCGA	115
***VEGFA***	TGCAGATTATGCGGATCAAACC	TGCATTCACATTTGTTGTGCTGTAG	81
***VEGF R1***	CAGGCCCAGTTTCTGCCATT	TTCCAGCTCAGCGTGGTCGTA	82
***VEGF R2***	CCAGCAAAAGCAGGGAGTCTGT	TGTCTGTGTCATCGGAGTGATATCC	87
***ACT-B***	CAAGATCATTGCTCCTCCTG	ATCCACATCTGCTGGAAGG	90
***TNF*** **α**	GACAAGCCTGTAGCCCATGT	CTCTGATGGCACCACCAACT	132
***E-Cadherin***	TGCTCGTGTTTGACTATGAAGG	TGGTCTTTGTCTGACTCTGAGG	82

### Ethical considerations

The study was approved by the Ethics Committee of Iran University of Medical Sciences, Tehran, Iran (Code: IR.IUMS.FMD.REC.1400.147) and registered on the Iranian Registry of Clinical Trials (last updated: 27/01/2024). All participants signed a written informed consent form.

### Statistical analysis

SPSS 21 software (Statistical Package for Social Sciences version 21.0, Chicago, Illinois, USA) was used for analysis in this study. Also, the molecular experiment data was obtained from the QIAGEN web portal at GeneGlobe. Moreover, the Student's *t* test was employed to compare 2 groups with continuous numerical data, while the Chi-square test was utilized to compare groups with categorical variables. A p-value < 0.05 was considered statistically significant.

## 3. Results

**Figure 2:**
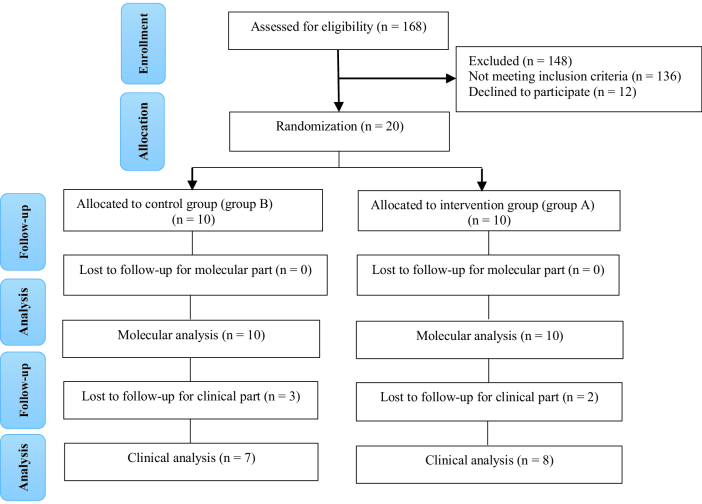
CONSORT diagram showing enrolled and included participants in the analyses.

Initially, 20 women with uRIF were eligible for inclusion in the study and were randomized to 2 groups (10 in each group). As shown in figure 2, for the primary outcome (molecular analysis), all endometrial samples of participants were evaluated (10 per group). Demographic information of the participants is shown in table II. For clinical outcome evaluation, the data of 2 women in the intervention group (group A) and 3 in the control group (group B) were excluded due to cancelation of ET. The reasons for ET cancelation in the intervention group were the risk of ovarian hyperstimulation syndrome (OHSS) and low ovarian response; for the control group (group B), the risk of OHSS, low quality of retrieved embryos, and low ovarian response were excluded (Table III). Finally, the clinical data of 15 participants in the 2 groups were analyzed. Participant recruitment began on June 20, 2021 and terminated on July 21, 2023, at the last newborn delivery.

### Demographic information

As indicated in table II, there was no significant difference between the intervention (group A) in terms of age, body mass index, duration of infertility, hormonal profile (AMH, LH, and FSH), or clinical outcome with control (group B) group.

### Clinical outcome

It should be noted that the fertilization and implantation rate, and chemical or clinical pregnancy rate between the 2 groups were not statistically significant. In addition, the 2 groups did not show any significant difference in terms of cancelation rate, incidence of blighted ovum, ectopic pregnancy, miscarriage rate, multiple pregnancies, or live birth rate (p > 0.05) (Table III).

### Global analysis of angiogenesis-related gene expression patterns

**Figure 3:**
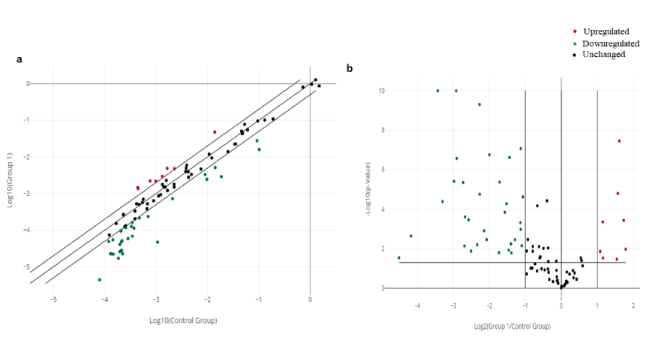
A) Scatter plot comparing the normalized expression of each gene on the human angiogenic growth factors PCR array between the control (group B) and intervention groups (group A). The diagonal line at the center shows unchanged gene expression, while the outer lines indicate the selected fold regulation threshold. Genes with data points beyond the outer lines are upregulated or downregulated. B) Volcano plot of isolates with significant gene expression alterations according to the log2 plot of the fold changes in gene expression. The horizontal line indicates the selected p-value threshold. The data are presented as fold changes. Student's t test was used.

**Figure 4:**
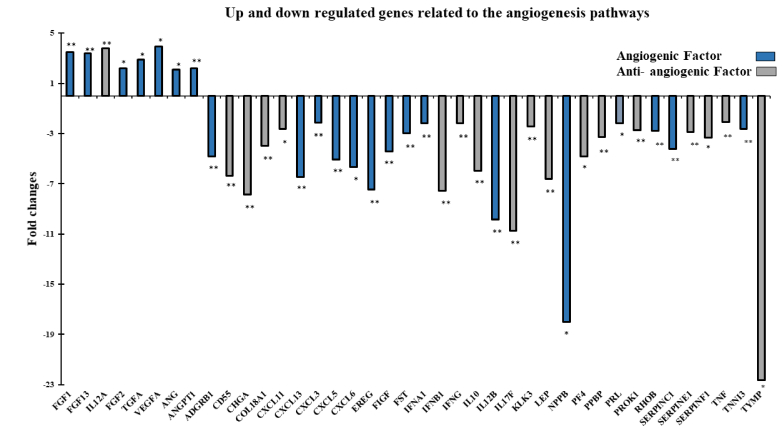
Up and downregulated angiogenic and antiangiogenic factors of endometrial samples from the intervention group (group A) compared to those from the control group (group B) (*p < 0.05, **p < 0.01). The data are presented as fold changes. Student's t test was used.

By combining the fold change results with the p-value statistical test results, we compared the genes that were overexpressed or underexpressed in the intervention group (group A) vs. the control group (group B), as shown in figures 3a, b. Analysis revealed a 47.6% difference in the expression of the 84 human angiogenic growth factor genes following ES, with 80% of these genes downregulated and 20% upregulated. All genes in the kit were classified into angiogenic or antiangiogenic factors according to their function in angiogenesis. As shown in figure 4, among the upregulated genes, *FGF1*, *FGF13*, *FGF2*, *TGFA*, *ANG*, *ANGPT1*, and *VEGFA* have angiogenic effects, while *IL12A* is an angiogenic inhibitory cytokine. Among the genes whose expression was downregulated after ES, *CXCL11*, *CXCL13*, *CXCL3*, *CXCL5*, *CXCL6*, *EREG*, *FIGF*, *FST*, *IL10*, *LEP*, *PPBP*, *PROK1*, *RHOB*, *TNF,* and *TYMP* (15 genes) have angiogenic functions. In contrast, 17 genes, including *ADGRB1*, *CD55*, *CHGA*, *COL18A1*, *IFNA1*, *IFNB1*, *IFNG*, *IL12B*, *IL17F*, *KLK3*, *NPPB*, *PF4*, *PRL*, *SERPINC1*, *SERPINE1*, *SERPINF1*, and *TNNI3*, are among the cytokines, chemokines, or growth factors included among the antiangiogenic factors.

### Data from confirmatory gene expression profiling via quantitative reverse transcriptase polymerase chain reaction

**Figure 5:**
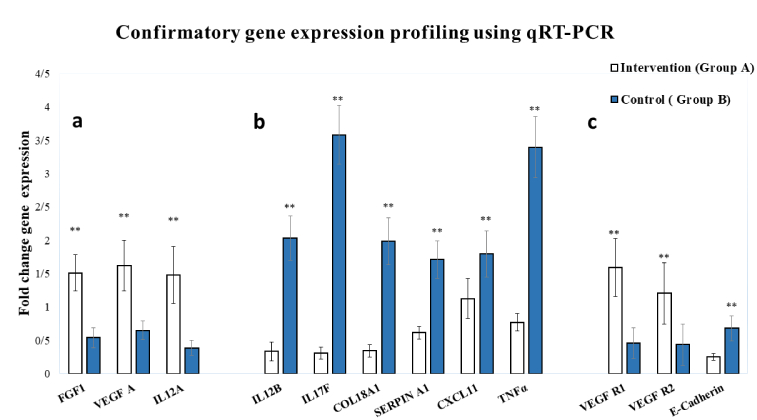
Gene expression profiling was confirmed via qRT-PCR to validate the data obtained from the PCR array. A) Upregulated genes in the intervention group (group A). B) Downregulated genes in the intervention group (group A). C) Data for 3 common key genes involved in endometrial angiogenesis and receptivity. *P < 0.05, **P < 0.01. Student's *t* test.

To validate the PCR array data, 9 representative genes whose expression significantly increased or decreased by more than 2-fold were selected (p < 0.05) for qRT-PCR analysis. The upregulated genes (*FGF1*, *VEGFA*, and *IL12A*) were confirmed to be significantly more highly expressed in samples from endometrial scratch tissue than in those from control tissue (p < 0.01) (Figure 5a). The selected downregulated genes identified by PCR array (*IL12B*, *IL17F*, *COL18A*, *SERPINF1*, *TNF*α, and *CXCL11*) were also validated by qRT-PCR and were confirmed to be expressed at significantly lower levels (Figure 5b). As previously mentioned, 3 additional genes, *VEGFR1*, *VEGFR2*, and *E-cadherin*, were also evaluated via qRT-PCR. The data showed that *VEGFR1* and *VEGFR2* expression levels were significantly increased. In contrast, the mRNA expression level of *E-cadherin* decreased considerably in the endometrial tissue sample from the intervention group (group A) after scratching (p < 0.01) (Figure 5c).

**Table 2:** Demographic information of participants in the studied groups


**Variable**		**Intervention group (n = 10)**	**Control group (n = 10)**	**P-value**
Age (yr)*		33.50 ± 1.80 (32.5, 6)	32.90 ± 1.9 (31, 6)	0.49
BMI (kg/m^2^)*		24.13 ± 0.68 (23.47, 1.72)	23.7 ± 1.08 (22.75, 3.1)	0.36
Duration of infertility (yr)*		9.40 ± 2.7 (7, 9)	8.70 ± 2.05 (6.75, 6)	0.52
**Hormonal profile (day 3 of the menstrual cycle)**				
	**AMH (ng/ml)** *	2.85 ± 1.33 (1.75, 4.3)	2.31 ± 1.08 (1.25, 2.92)	0.34
	**LH (IU/L)** *	5.57 ± 2.36 (4.025, 8.2)	4.61 ± 1.68 (3.35, 4.6)	0.31
	FSH (IU/L)* * *	7.07 ± 1.52 (5.525, 4.7)	6.35 ± 1.63 (5.025, 4.7)	0.32
*The data are presented as the Means ± SD (median, interquartile range). Student's *t* test was used. BMI: Body mass index, AMH: Anti-Mullerian index, LH: Luteinizing hormone, FSH: Follicle-stimulating hormone				

**Table 3:** Clinical outcomes of group A and group B


**Variable**	**Intervention group (n = 10)**	**Control group (n = 10)**	**P-value**
Total dose of gonadotropin (IU)*	2126 ± 772 (1612.5, 2505)	1927 ± 790 (1481.25, 2400)	0.514
Duration of ovarian stimulation (day)*	11 ± 0.94 (10.75, 3)	11.1 ± 1.1 (10.75, 4)	0.83
Number of retrieved oocytes*	6.70 ± 3.4 (5.75, 14)	7.4 ± 4 (5, 14)	0.68
**Metaphase II** * * oocytes* * *	4.90 ± 2.7 (2.75, 10)	4.8 ± 4.40 (2, 14)	0.90
Fertilization rate*	74 ± 28.7 (67.5, 100)	70 ± 31.2 (47.5, 100)	0.76
**Embryo development rate** *	86.11 ± 13.81 (83.33, 33.33)	90.27 ± 17.43 (100, 50)	0.79
Number of embryos*	3.3 ± 2.1 (2, 8)	2.9 ± 1.5 (2, 5)	0.60
Implantation rate** * *	7/16 (43.75)	4/14 (28.57)	0.12
ET cancelation** * *	2/10^1^	3/10^2^	0.45
**Chemical pregnancy rate/ET** **	6/8 (75)	4/7 (57.1)	0.40
Clinical pregnancy rate/ET**	6/8 (75)	4/7 (57.1)	0.40
**Blighted ovum/ET** **	1/8 (12.5)	0/7	0.44
**Ectopic pregnancy/ET** **	1/8 (12.5)	0/7	0.44
**Miscarriage rate/ET** **	1/8 (12.5)	1/7 (14.2)	0.39
Multiple pregnancy (Twin)**	1/6 (16.6)	0/4	0.44
**Live birth rate/ET** **	4/8 (50)	3/7 (75)	0.84
*The data are presented as the Mean ± SD (median, interquartile range), Student's *t* test. **The data are presented as n (%), Chi-square test. ET: Embryo transfer. ${\;}^{1}$ Case 1: High ovarian response (risk of OHSS). Case 2: Low ovarian response. ${\;}^{2}$ Case 1: High ovarian response (risk of OHSS). Case 2: Low quality of obtained embryos. Case 3: Low ovarian response			

## 4. Discussion

The results of our study revealed notable alterations in the expression levels of angiogenic and antiangiogenic genes following ES intervention (group A) compared to those in the control group (group B). The observed downregulation of a majority of angiogenic genes post-ES suggested a complex regulatory response within the endometrial microenvironment. Interestingly, a subset of angiogenic factors, including *FGF1*, *FGF2*, *VEGFA*, and *ANGPT1*, exhibited upregulation, potentially indicating a selective enhancement of angiogenic processes.

Among the upregulated genes in our study, *FGF1*, *FGF2*, and *FGF13*, which belong to the *FGF* family, promote the formation of blood vessels that improve endometrial trophoblastic interactions and, consequently, implantation. Another study also showed that the expression of these genes increased at 5–7 days postovulation during implantation (20), while the expression of *FGF1* was significantly reduced in the RIF endometrium (21). It appears that ES can compensate for and induce *FGF* expression, thereby overcoming this alteration in RIF women. The increase in *VEGF* (or its isoform, *VEGFA*) gene expression following ES likely arises from a multifaceted interplay of cellular and molecular responses. ES induces physical microtrauma to endometrial tissue, initiating a wound-healing response in which VEGF, is pivotal for angiogenesis, aids in repairing and regenerating damaged blood vessels and tissues. Moreover, ES may create a localized hypoxic environment, promoting the activation of hypoxia-inducible factor 1-alpha, which in turn upregulates *VEGF* expression (22).

Since RIF was recently stratified based on molecular signatures, it is important to correlate these molecular signatures with the clinical consequences of RIF. RIF is caused by at least 2 molecular events: asynchrony in molecular pathways and/or disruption of these pathways during the short term of the WOI (23). One of the main endometrial alterations is its ability to induce angiogenesis in WOI (3). The most critical primary endometrial response to scratching is angiogenesis (24, 25). The mechanical injury induced by scratching is recognized as a crucial factor in initiating a reparative reaction within endometrial tissue. This process entails the mobilization of regenerative cells and the secretion of signaling molecules, which contribute favorably to the enhancement of endometrial readiness. Hemostasis, inflammation, and proliferation are all involved in the healing process that follows scratching, during which angiogenesis occurs during the proliferation phase (12, 24).

Among all the factors that mediate angiogenesis during implantation, *VEGF*, a potent angiogenic factor, plays an essential role in various physiological and pathological conditions in females (6). Interestingly, its corresponding receptors, *VEGFR-1* and *VEGFR-2*, which mediate the vascular hyperpermeability required for blastocyst implantation, were found to be expressed in the endometrium at peri-implantation stages (6, 26). In addition to its role in angiogenesis, *VEGF* acts as an immune modulator that mediates maternal immunological tolerance by recruiting or activating macrophages and uterine natural killer cells during embryo implantation. This macrophage homing to the decidua contributes to the establishment and maintenance of pregnancy, while macrophage phenotype switching by altering *VEGF* function is associated with different pregnancy disorders (6, 27). The present study showed that the *VEGFA* gene's and both receptors' expression increased significantly after scratching compared with that in the control group (group B) (Figures 4, 5a). This result is consistent with another study by Liang et al., who showed that a scratching-induced increase in *VEGF* in women with unexplained subfertility (25). Furthermore, ES may stimulate the release of growth factors such as *FGF*s and *TGF- β *, which are known to promote tissue repair and angiogenesis, further enhancing *VEGF* expression. Together, these mechanisms underscore the complex orchestration of tissue repair, angiogenesis, and endometrial remodeling in response to ES.

The temporal-spatial expression of *VEGF* should be highly controlled during and after the embryo implantation timeline; otherwise, its overexpression results in later pregnancy complications, such as pregnancy loss and recurrent abortion (28, 29). Therefore, it is essential to regulate downstream *VEGF* pathways by balancing inhibitors or activating factors. For this purpose, we took a closer look at genes involved in angiogenesis pathways and then classified them into 2 subgroups based on their roles as either angiogenic or antiangiogenic factors. Because the implantation rate improved, we hypothesized that only genes involved in angiogenesis could have played a role in achieving this result. However, as shown in the results section (Figure 4), either angiogenic or antiangiogenic factors were up- or downregulated, it was found that scratching induces 2 opposing forces-angiogenesis and antiangiogenic-with the net force of boosting implantation.

Degradation of the extracellular matrix is a critical step in the early stages of angiogenesis (30). Interestingly, the observed decrease in *E-cadherin* expression following scratching in the present study (Figure 5c) may be attributed to this process, as downregulation of this cell-cell adhesion molecule could create a conducive environment for neovascularization. While some studies have reported decreased *E-cadherin* expression in RIF women, we hypothesize that its expression must be carefully balanced during and after implantation (31, 32). Specifically, the expression of this gene may decrease during neovascularization but increase during implantation, suggesting a finely regulated timeline for its expression.

On the other hand, several antiangiogenic factors-such as important arms of neovascularization for embryo implantation, were altered after scratching (Figure 4). However, their angiogenic inhibitory mode is solely one side of the coin. These factors contribute to successful implantation in normal pregnancies, while a high production of these factors is detrimental to embryo survival. Furthermore, high expression of *IL10*, *IFNG*, and *TNF-*α (33, 34) may have adverse effects on the endometrial receptivity and apposition/adhesion phases of embryo implantation (35, 36). For instance, some pregnancy complications have been linked to an increase in the *IFNG* levels. Since, studies have shown that *IFN* prevents the implantation and maintenance of pregnancy in the preimplantation period by reducing the production of *GM-CSF* and other cytokines, which promote the growth and maturation of blastocysts (34, 37).

Scheliga et al. conducted a proteomic analysis and demonstrated that ES probably affects immune response pathways and cytoskeleton formation, which has potential implications for an increase in endometrial receptivity. Following ES, there was an increase in the abundance of proteins associated with immune response and cytoskeleton regulation, while conversely, a decrease in the abundance of proteins involved in actin cytoskeleton regulation and cellular processes such as intracellular transport, apoptosis, and autophagy was noted. These changes may enhance embryo implantation (11). At the time of embryo implantation, immune cells modulate critical factors such as *TGF*, *FGFs*, *VEGF*, and IGFs in the human endometrium. Therefore, they are believed to be involved in the reconstruction of blood vessels and the modulation of vascular alterations during implantation and pregnancy (3, 38).

Notably, in the present study, ES was performed during the proliferative phase of the menstrual cycle in the intervention group (group A), so molecular or gene alterations in the secretory functions of immune cells are derived from the time of scratching when the nature of immunologic and angiogenic responses differs from that of the secretory phase. Since the menstrual cycle is a highly dynamic process and chronological sequences of molecular expression change every day or even every hour, the day of endometrial biopsy is an important variable for interpreting the results. These fluctuations underpin the need for later endometrial preparation for implantation (39).

Even though participants in the current study were divided into 2 groups for molecular investigation, all of them were biopsied in the secretory phase; therefore, the clinical outcome was attained through ES in all the enrolled women. In our study, the total implantation rate was 36.6% in women who had previously failed IVF/ICSI treatments. In addition, 46.6% of all pregnancies/ETs reached full term; for example, 7 live births per 15 infertile women had beneficial effects from scratching, which is consistent with the findings of other studies of unexplained women; however, to draw a more comprehensive conclusion about the clinical outcome, a larger sample size is needed (40).

## 5. Conclusion

According to the findings of this study, ES appears to balance angiogenic and antiangiogenic processes within the endometrium of individuals with uRIF, ultimately leading to favorable effects that support implantation.

##  Data availability

The data supporting the findings of this study are available upon reasonable request from the corresponding author.

##  Author contributions

S.A., F.M., F.A., Z.Z., M.B., F.Gh. and R.A. designed the study. S.A. conducted the research. S.A. evaluated and analyzed the results of the study. Furthermore S.A., E.H., and Kh.A. drafted the manuscript and reviewed the article. M.B. and R.A. supervised the study. All the authors approved the final manuscript and took responsibility for the integrity of the data.

##  Conflict of Interest

The authors declare that there is no conflict of interest.
